# Inhibition of 5-lipoxygenase downregulates stemness and kills prostate cancer stem cells by triggering apoptosis via activation of c-Jun N-terminal kinase

**DOI:** 10.18632/oncotarget.13422

**Published:** 2019-01-11

**Authors:** Sivalokanathan Sarveswaran, Nadimpalli R.S. Varma, Shravan Morisetty, Jagadananda Ghosh

**Affiliations:** ^1^ Vattikuti Urology Institute and Josephine Ford Cancer Center, Henry Ford Health System, Detroit, MI 48202, USA

**Keywords:** 5-lipoxygenase, c-Myc, prostate cancer stem cells, MK591, apoptosis

## Abstract

The cancer stem cell (CSC) concept suggests that neoplastic clones are maintained exclusively by a rare group of cells possessed with stem cell properties. CSCs are characterized by features that include self-renewal, pluripotency and tumorigenicity, and are thought to be solely responsible for tumor recurrence and metastasis. A hierarchically organized CSC model is becoming increasingly evident for various types of cancer, including prostate cancer. The CD44 ^(+)^, CD133 ^(+)^ cell subpopulations were isolated from human prostate tumors which exhibit stem-like properties showing therapeutic-resistance, capacity of self-renewal, and exact recapitulation of the original tumor *in vivo*. Thus, an important challenge is to find measures to eliminate these cancer stem cells, which will stop tumor growth and prevent disease-recurrence. However, knowledge about molecular features critical for the survival of prostate cancer stem cells (PCSC) is meager. Here we report that inhibition of 5-lipoxygenase (5-Lox) by shRNA or MK591 dramatically kills PCSC by inducing apoptosis, suggesting that 5-Lox plays an essential role in the survival of PCSC. Interestingly, MK591 treatment decreases protein levels and inhibits transcriptional activities of Nanog and c-Myc. Since Nanog and c-Myc play important roles as stemness factors, our findings indicate that the 5-Lox activity plays a causal role in maintaining prostate cancer stemness via regulation of Nanog and c-Myc, and suggest that further exploration of 5-Lox-mediated signaling in PCSC may lead to development of novel, target-based, durable strategies to effectively block development and growth of prostate tumors, and prevent prostate cancer recurrence.

## INTRODUCTION

The concept of cancer stem cell propounds that a small fraction of cells in tumors are capable of self-renewal and to differentiate into various types of tumor cells, and are responsible for tumor formation, growth, progression and recurrence, while the majority of cells that make up bulk of the tumors are non-tumorigenic end cells [1–3]. The discovery of cancer stem cells has redesigned the concept of therapy of various types of cancer, because the “non-stem” cancer cells in a tumor can be destroyed by conventional therapies (e.g., radiation, surgery, chemotherapy), but these approaches are rarely successful in eliminating cancer stem cells [4–7]. Thus, the goal of current cancer therapy should be to develop strategies to destroy these resistant cancer cells endowed with stemness. Though our knowledge of CSCs and their micro-environmental niche is still a relatively new field, recent understanding about CSC's dependence on critical survival and self-renewal signaling makes these regulatory pathways ready for development of novel therapeutic strategies. Cancer stem cells are a rare subset of cells in a tumor which are capable of eluding conventional treatment regimen and eventually regenerate tumors. Cancer stem cells can be extracted from a tumor based on molecular markers, and transplanted to form new tumors, from which the same tumor-propagating cells can again be isolated and re-transplanted [8]. This view puts forth the hypothesis that the vast majority of cells in a tumor undergo differentiation after originating from cancer stem cell and lose their self-renewal potential, and thus, cannot contribute to tumor formation and perpetuation. The cancer stem cell concept has inspired many due to the potential of providing more durable and broadly applicable cancer cures by locating and targeting the tumor's most notorious cells. However, many of the molecular features that regulate stemness and survival of cancer stem cells for their seemingly infinite and incessant proliferative capabilities are just now beginning to be appreciated.

Cancer cells with stem-like properties have been identified in prostate tumors and characterized to be the population responsible for generating the diverse cell types within prostate tumors [8–12]. CSCs are also regarded as the seeds for therapeutic resistance, tumor recurrence and metastasis, and are recognized by a number of defined markers, such as they are positive for CD133 and CD44, and express high levels of integrin alpha2beta1 [13–17]. Recent identification of pluripotency markers (Nanog, c-Myc, Sox2) in prostate cancer stem cells and characterization of their unique molecular properties have opened up possibilities about how they can be targeted for more effective therapies. However, many of the stemness as well as tumorigenic markers are also present in other body cells, including normal stem cells [18, 19]. Thus, finding the root cause of therapeutic failures and targetable unique molecular properties need further characterization of prostate cancer stem cells. Most current therapies are directed to the majority of tumor mass consisting of fast-growing, differentiated cells but not the relatively slow-growing and resistant cancer stem cells which may underlie as a mechanism of why the cancer is not permanently eradicated with standard chemotherapy. The concept of CSC claims that cancers are driven by a set of stem cells in the same way as adult organs are maintained by dedicated tissue-specific stem cells [18]. Provoked by this new concept, a lot of excitement is now directed to go right after the root cause of the tumor and target those stem cells and eradicate cancer permanently. However, the destruction of prostate cancer stem cells should include measures that will selectively and systematically affect functions of critical molecular markers responsible for the maintenance, growth and differentiation of PCSCs. Thus, our approach was directed to examine the role of a newly-characterized mechanism, which plays important roles in the survival and proliferation of prostate cancer cells but not of normal cells [20–26], in the maintenance of stemness and survival of PCSC.

Our strategy was to utilize an established, clinically-relevant model of human PCSCs to interrogate the mechanisms of stem cell vulnerabilities and to find effective measures for controlling the population of PCSC which express both pluripotency markers (Nanog, c-Myc and Sox2) as well as the tumor-progenitor markers (CD44, CD133, and ALDH1) [27–29]. Especially for this work, our goal was to explore and characterize the role of a fatty acid metabolic pathway in human prostate tumor-derived stem cells, to see whether it could be used as a mechanism-based target to control PCSC. Interestingly, most non-cancer parenchyma body cells, including normal stem cells, do not express 5-Lox under normal conditions, presumably because of hyper-methylation of the 5-Lox promoter [30, 31]. Moreover, 5-Lox knock-out mice have been developed which grow normally without any abnormalities and are fertile with normal litter size, supporting the concept that targeting 5-Lox may turn-out to be a viable strategy to effectively eliminate PCSC without affecting most other normal body cells, and thus will not trigger overt toxicity to general health. We found that inhibition of 5-Lox downregulates Nanog, c-Myc and Sox2 and blocks sphere-formation, which suggests that the stemness characteristics in these cells are vulnerable to inhibition of 5-Lox activity. We also found that MK591, a specific 5-Lox inhibitor, dramatically reduces the viability and colony-forming abilities of PCSC, and induces c-JNK-dependent apoptosis. These findings indicate that 5-Lox activity plays an important role in the survival of PCSC, and suggest that an effective, long-term curative therapy of prostate cancer is possible by targeting 5-Lox with suitable agents to eliminate PCSC via induction of apoptosis.

## RESULTS

### Flow chart for isolation and characterization of prostate cancer stem cells from patient-derived tumors

**Figure 1:**
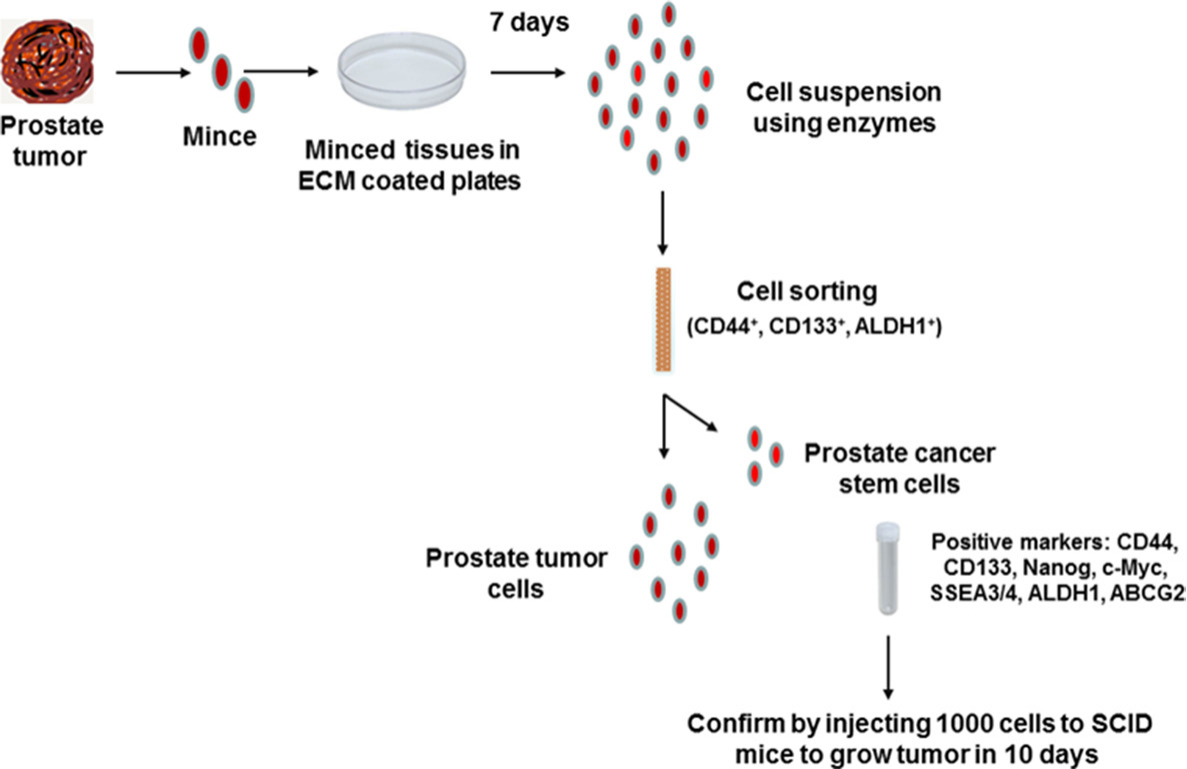
Flow chart of isolation and characterization of prostate cancer stem cells from human tumors Human prostate tumor tissues were finely minced with scalpel and placed on ECM (E36103-3PD)-coated plates in complete growth medium (M36103-30S) and incubated at 37°C in the incubator for seven days. Then the tissues were lysed in protease enzyme solution to make single cell suspension and sorted by Flow Cytometry using indicated markers. Positive cells were cultured in non-differentiating complete growth medium for expansion of cells and finally tested for tumorigenicity using 1000 cells per injection site in SCID mice to develop subcutaneous tumors in ten days.

A flow chart depicting the process of isolation of prostate cancer stem cells from patient prostate tumors is shown in Figure 1. These cells were rigorously tested for expression of stemness markers as well as for their high tumorigenicity by injecting 1000 cells per injection site in SCID (Severe Combined Immuno-Deficient) mice for quality control (Courtesy: Dr. Jay Sharma, Celprogen. com, Torrance, CA). These cells were cultured on ECM (E36103-3PD)-coated plates in complete growth medium (M36103-30S) and incubated at 37°C in the incubator for expansion to get the desired numbers of cells for experiments. Positive markers for stemness and tumorigenicity of these cells were extensively characterized as a part of this study (Please see below).

### 5-lipoxygenase is heavily expressed in prostate cancer stem cells and plays a critical role in their survival

**Figure 2:**
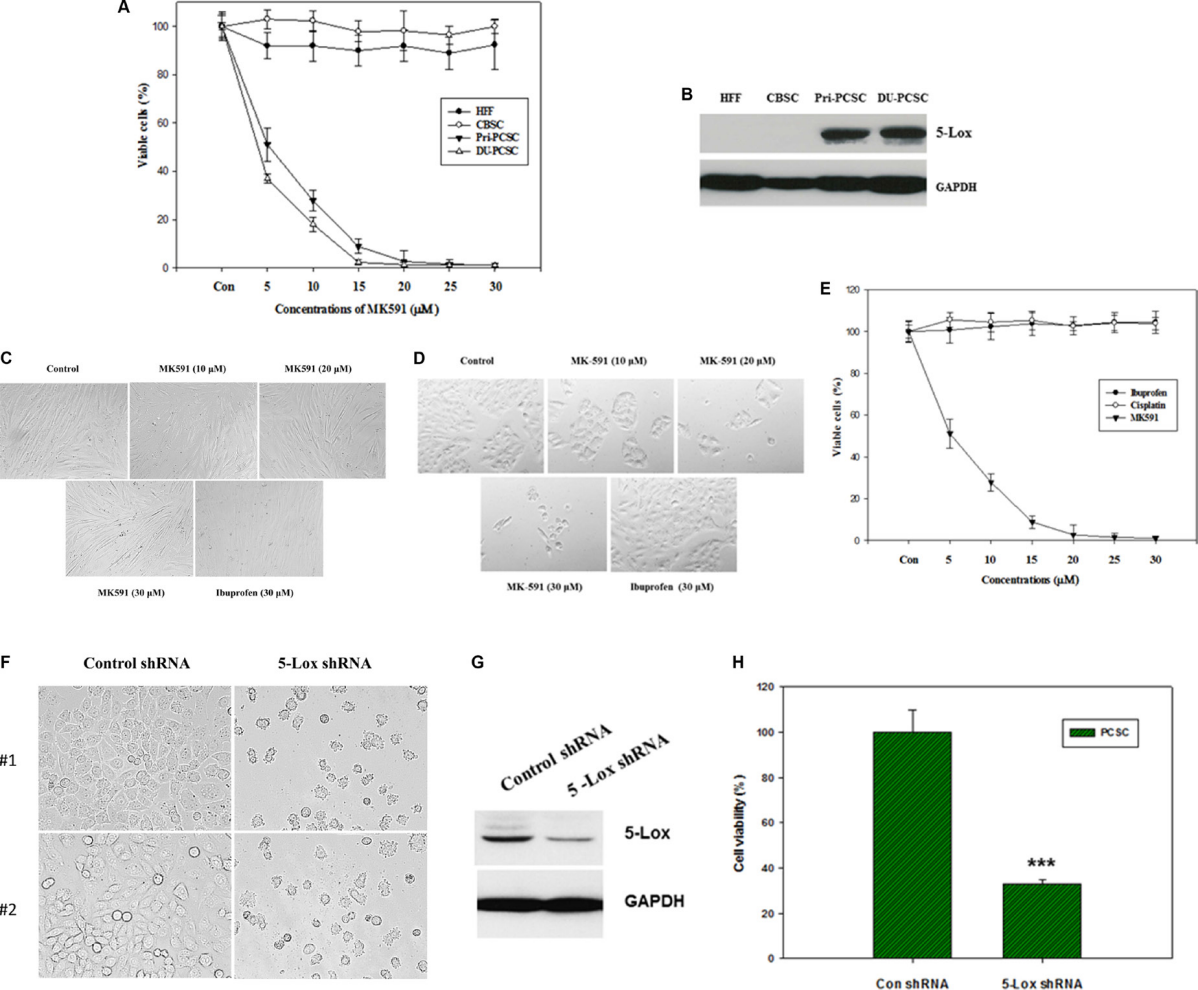
MK591 decreases viability of prostate cancer stem cells, but not of non-cancer cells or normal stem cells (**A**), Human tumor-derived primary prostate cancer stem cells (Pri-PCSC) and DU145 cell line-derived cancer stem cells (DU-PCSC) were plated in 96 well tissue culture plates in complete growth medium supplemented with 10% serum. Normal human foreskin fibroblasts (HFF) and cord blood stem cells (CBSC) were also plated in serum-supplemented complete growth medium in parallel, Then, the cells were treated with varying doses of MK591, and the plates were further incubated for 72 hours at 37°C in the CO_2_ incubator. After incubation, cell viability was measured by MTS assay (Promega Corp, Wisconsin, MD). In (**B**), protein level of 5-lox was detected by Western blot. (**C**) and (**D**) show morphological alterations and culture density of normal HFF cells and PCSC as detected by microscopy. In (**E**) sensitivity of prostate cancer stem cells to different compounds was tested by MTS assay. Data presented as mean values of quadruplicate determination of each data point ± Standard Error. Note: While MK591 effectively killed PCSC, ibuprofen (a cyclooxygenase inhibitor) and cisplatin were found ineffective. (**F**–**H**) show morphology, level of 5-Lox protein, and viability of PCSC after treatment with shRNA against 5-Lox.

Earlier we reported that under standard culture condition prostate cancer cells express 5-Lox but non-cancer cells do not, and that the 5-Lox activity plays a critical role in the survival of prostate cancer cells [20–26]. However, nothing was known about whether 5-Lox plays any role in the survival of prostate cancer stem cells. To explore a possible role of 5-Lox in prostate cancer stem cells, we observed that treatment with MK591, which specifically inhibits 5-Lox activity by binding with FLAP or 5-lipoxygenase-activating protein [32–36], dramatically decreases the viability of PCSCs in a clear dose- and time-dependent manner (Figure 2A–2E). Similar changes were also found when PCSCs were treated with 5-Lox shRNA, confirming a role of 5-Lox in the viability of these cells (Figure 2F–2H). Interestingly, MK591 does not affect the viability of normal cells or non-cancer stem cells under similar experimental conditions. To understand the basis of this differential sensitivity to 5-Lox inhibitors, we found that in contrast to prostate cancer stem cells, normal fibroblasts or the non-cancer stem cells do not express 5-Lox. These findings suggest that specific inhibitors of 5-Lox may turn out to be useful to specifically target PCSCs without harming non-cancer body cells. Though we are intrigued by this novel and unexpected observation, downstream signaling mechanisms which mediate the regulation of prostate cancer stem cell survival by 5-Lox activity are yet to be fully understood.

### Inhibition of 5-Lox downregulates stemness in prostate cancer stem cells

**Figure 3:**
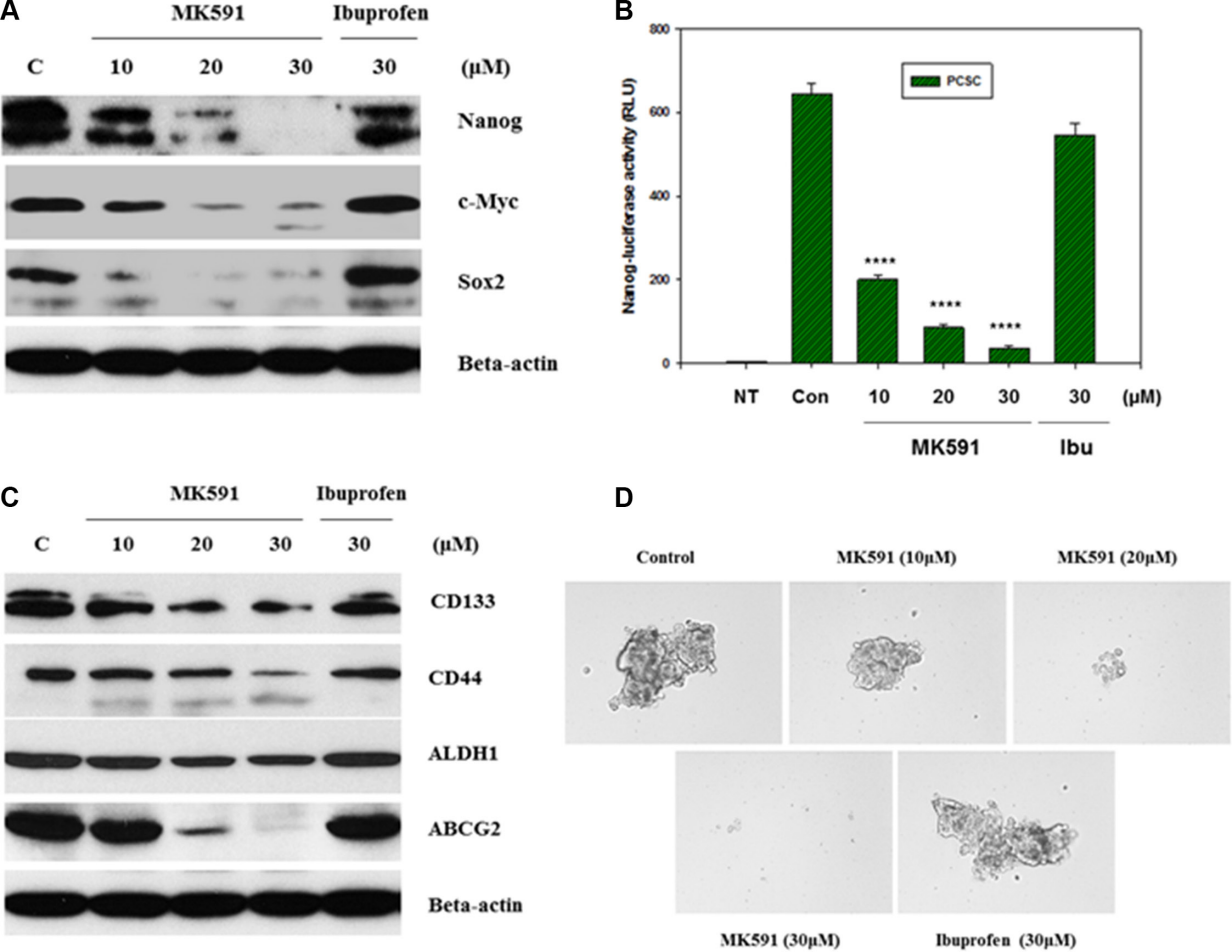
Inhibition of 5-Lox downregulates stemness in PCSC In (**A**), PCSCs (3 × 10^5^ per plate) were plated in complete growth medium supplemented with 10% serum in 60 mm diameter plates and allowed to grow for 48 hours. On the day of experiment, the spent culture medium was replaced with 2 ml fresh RPMI medium and the cells were treated with MK591 or ibuprofen at 37°C for 24 hours as indicated. Control cells were treated with solvent only (0.2% DMSO). At the end of incubation period, cells were harvested and lysed. Protein levels in treated and untreated cells were detected by Western blot. (**B**) shows transcriptional activity of Nanog after treatment with varying doses of MK591 in Nanog-luciferase transfected PCSC. In (**C**), protein levels of adult stem cell markers are shown with or without treatment with MK591. (**D**) shows effect of MK591 on sphere formation by PCSC in ultra-low attachment plates in complete growth medium.

Stemness in normal as well as cancer stem cells is maintained by a list of transcription factors such as, Nanog, c-Myc, Oct-4 and Sox-2 [13–17]. These stemness factors have also been well-characterized in PCSC and found to play important roles in the maintenance of stemness. However, details of their upstream and downstream regulations have yet to be fully characterized which may yield valuable information about manipulating them for long-term cancer control. Interestingly, we found that inhibition of 5-Lox dramatically downregulates the protein levels of Nanog, c-Myc and Sox2, indicating that the 5-Lox activity plays an important role in the regulation of stemness in PCSCs (Figure 3A). The decrease in protein level of Nanog was also found to be well-correlated with a decrease in its transcriptional activity (Figure 3B). Recently, we reported that inhibition of 5-Lox downregulates expression and function of c-Myc in prostate cancer cells involving inhibition of Stat3-mediated transcription [25, 26]. Moreover, we observed that inhibition of 5-Lox downregulates CD44, CD133, ALDH1 and ABCG2 which are characterized to be cancer stem cell- or tumorigenicity-markers in adult stem cells (Figure 3C). Though mechanistic details of the regulation of stemness factors by the activity of 5-Lox in the biology of PCSC are yet to be understood, our findings show a possible molecular connection between metabolism of arachidonic acid through 5-Lox and the maintenance of stemness in prostate cancer stem cells. Formation of spheres in low-attachment plates is a standard characteristic of cancer stemness. Interestingly, we found that MK591 treatment dramatically inhibits the sphere-forming ability of PCSCs in a clear dose-dependent manner, suggesting that the 5-Lox activity is critical for maintaining stemness in these cells (Figure 3D).

### MK591 destabilizes mitochondria and induces apoptosis in prostate cancer stem cells

**Figure 4:**
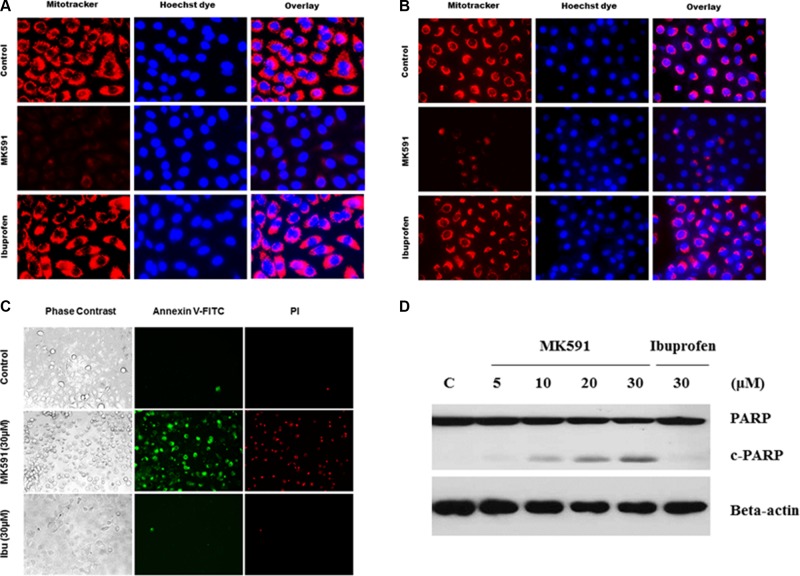
Inhibition of 5-Lox triggers mitochondrial permeability transition and induces apoptosis in PCSCs In (**A**) and (**B**), PCSC and DU-PCSC (3 × 10^5^ per plate) were plated in complete growth medium supplemented with 10% serum in 60 mm diameter plates and allowed to grow for 48 hours. On the day of experiment, the spent culture medium was replaced with 2 ml fresh RPMI medium and the cells were treated with MK591 as described in Figure 2. Then the cells were treated either with MK591 (30 μM) or ibuprofen (30 μM) for 24 hours. Then, the cells were stained with mitotracker-red for 60 minutes, counter-stained with Hoechst 33342 dye (blue), and observed under a fluorescent microscope (**C**). Membrane lipid asymmetry was detected by Annexin V binding. At the end of incubation period, cells in binding buffer were treated with FITC-labeled annexin-V and propidium iodide, and were observed under microscope at 20×. In (**D**), at the end of incubation, cells were lysed and cleavage of PARP was detected by Western blot.

Based on previous observations of a critical role of 5-Lox activity in prostate cancer stem cell survival, we wanted to explore downstream signaling mechanism(s) regulated by 5-Lox using MK591, a specific inhibitor of 5-Lox activity. We found that MK591 dramatically induces mitochondrial-membrane depolarization, which is indicated by the loss of permeability barrier of mitotracker-red (Figure 4A, 4B). Externalization of phosphatidylserine is a hall-mark of apoptotic cell death. Thus, we wanted to examine whether prostate cancer stem cells also externalize phosphatidylserine when treated with MK591. We observed that prostate cancer stem cells show distinctly positive binding with annexin-V when treated with MK591, indicating externalization of phosphatidylserine to the cell surface (Figure 4C). We also observed that when prostate cancer stem cells were treated with MK591, these cells showed severe morphological alteration in a time-dependent manner. Characteristic caspase-mediated cleavage of poly-ADP-ribose polymerase (PARP) is another indicator of apoptosis, which was also observed in PCSC when treated with MK591 (Figure 4D), suggesting that inhibition of 5-Lox kills prostate cancer stem cells by triggering apoptosis.

### Inhibition of 5-Lox triggers apoptosis in prostate cancer stem cells via activation of c-Jun N-terminal kinase

**Figure 5:**
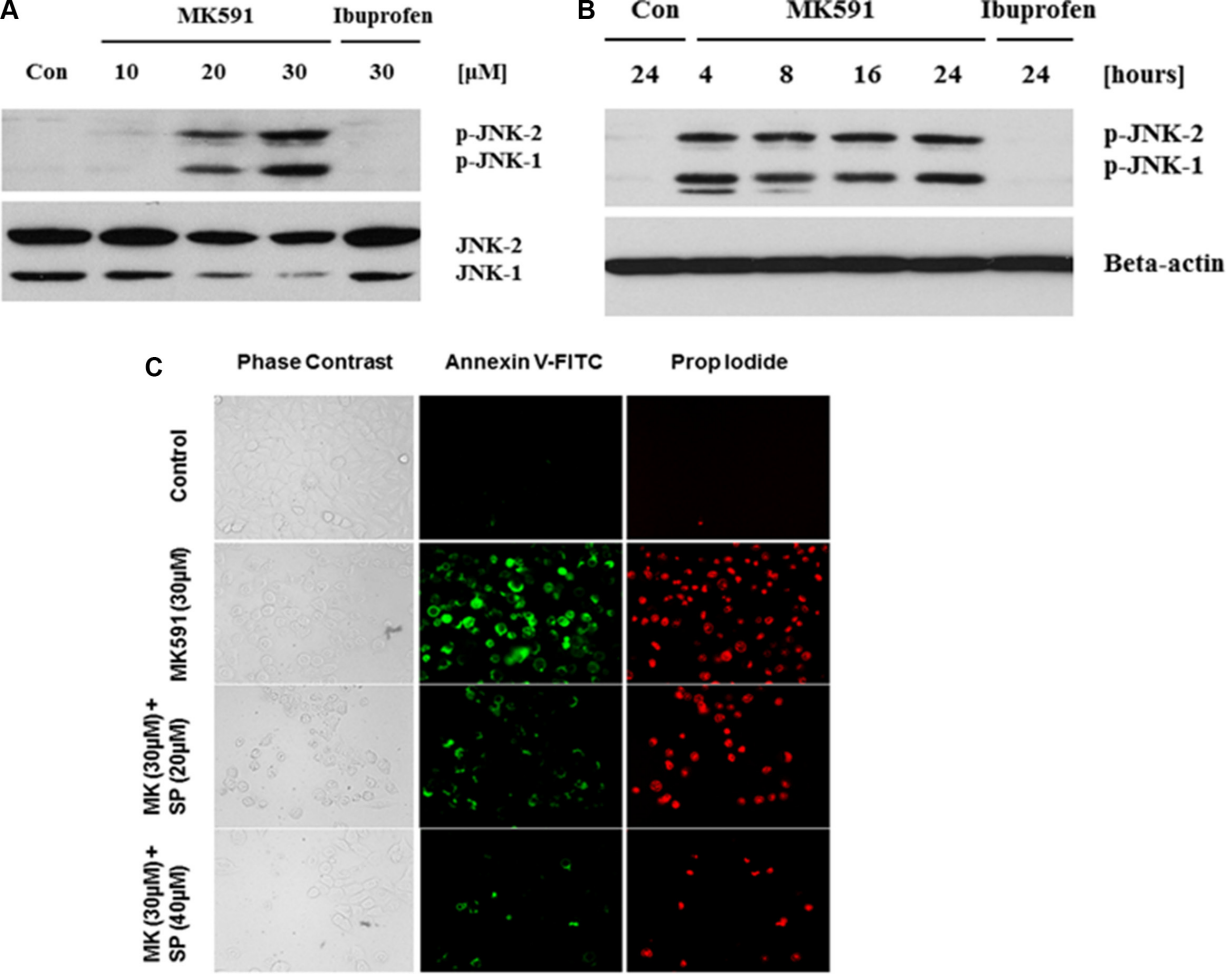
MK591 treatment triggers activation of c-Jun N-terminal Kinase which plays a critical role in apoptosis PCSCs (3 × 10^5^ per plate) were plated in complete growth medium supplemented with 10% serum in 60 mm diameter plates and allowed to grow for 48 hours. On the day of experiment, the spent culture medium was replaced with 2 ml fresh RPMI medium and the cells were treated with MK591 as described in Figure 2 for 24 hours. In (**A** and **B**), dose and time-dependent changes in phosphorylation of c-JNK by MK591 treatment were detected by Western blots using phospho-specific antibodies. In (**C**), cells were pre-treated with c-JNK inhibitor (SP = SP600125) for 30 minutes before treatment with MK591, and cell death was detected by staining with Annexin V-FITC as in Figure 4 above.

Previously we have addressed the mechanism behind 5-Lox inhibition-induced apoptosis-triggering in prostate cancer cells, which revealed the involvement of c-Jun N-terminal Kinase and caspase-3 [22]. To explore the underlying mechanism in 5-Lox inhibition-induced apoptosis in PCSC, we found that inhibition of 5-Lox triggers rapid and robust activation of c-Jun N-terminal kinase in a dose- and time-dependent manner (Figure 5A, 5B). Further analysis revealed that the MK591 treatment-induced apoptosis in PCSC is inhibited when the cells were pretreated with an inhibitor of c-JNK (SP600125), suggesting that 5-Lox inhibition-induced apoptosis in PCSCs is dependent on c-JNK activity (Figure 5C). Interestingly, cells treated with ibuprofen (a cyclooxygenase inhibitor) did not show any signs of apoptosis, suggesting specific effects of 5-Lox inhibition to induce apoptosis in prostate cancer stem cells.

### Inhibition of 5-Lox induces apoptosis in prostate cancer stem cells via downregulation of PKCε, but without inhibition of Akt or ERK

**Figure 6:**
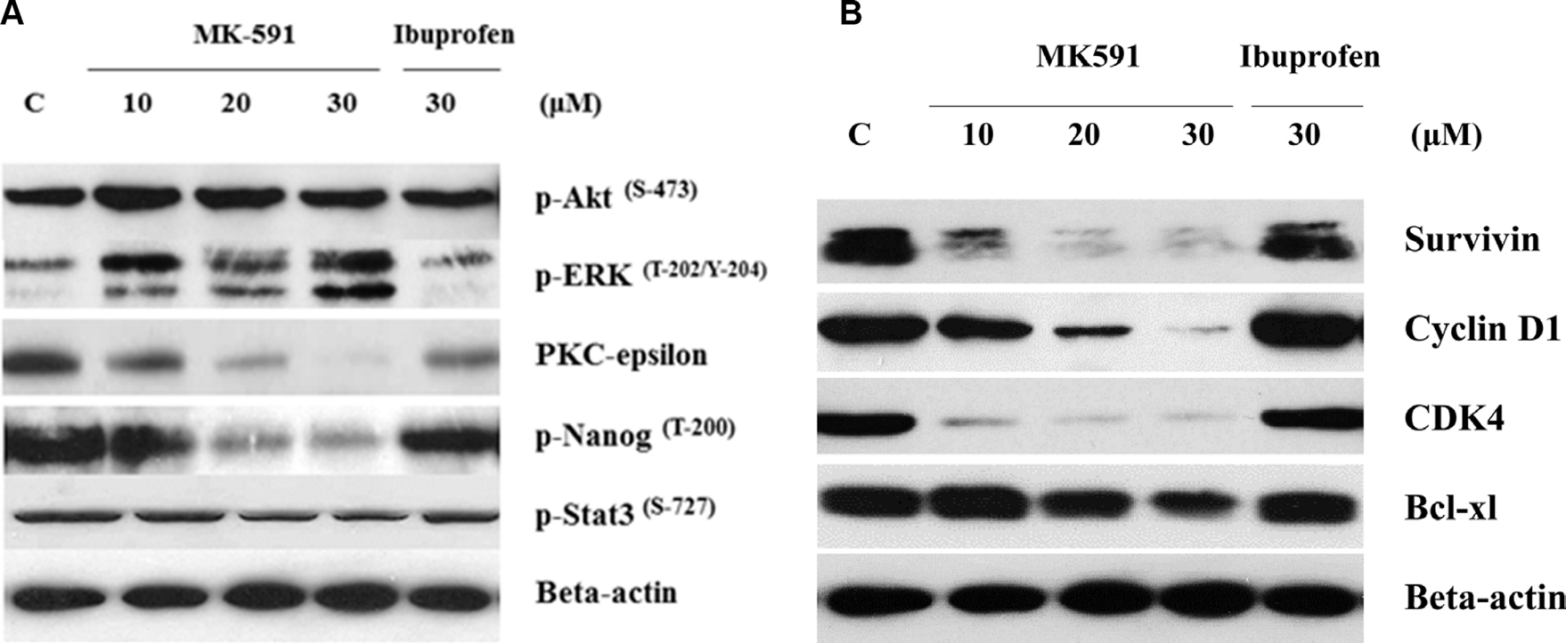
5-Lox inhibition-induced apoptosis in prostate cancer stem cells involves inhibition of PKCε, but not Akt or ERK PCS cells (3 × 10^5^ per plate) were plated in complete growth medium supplemented with 10% serum in 60 mm diameter plates and allowed to grow for 48 hours. On the day of experiment, the spent culture medium was replaced with 2 ml fresh RPMI medium and the cells were treated either with MK591 (10–30 μM) or ibuprofen (30 μM) for 24 hours. Control cells were treated with vehicle only (0.2% DMSO). At the end of incubation, cells were harvested and proteins were separated in 12% SDS-PAGE. In (**A**), protein levels of PKC-epsilon and phosphorylations of Akt-pSer^473^, ERK-pThr^202/Y204^, Nanog-pThr^200^ and Stat3-pSer^727^, and in (**B**) protein levels of survivin, cyclin D1, CDK4 and Bcl-xl were detected by Western blot. Data show a representative of three independent experiments with similar results.

How inhibition of 5-Lox triggers apoptosis in PCSC is an intriguing question. We addressed this by examining the effect of 5-Lox inhibition on well-characterized survival-regulating kinases. Previously, we reported that inhibition of 5-Lox induces apoptosis in prostate cancer cells without inhibition of the Akt, or ERK, but via a rapid decrease in the membrane-localization as well as enzymatic activity of PKCε [23, 37]. Involvement of PKCε has been reported to promote survival and decrease apoptosis in a variety of other cells [38–40]. To explore the kinase responsible for this mechanism of cell death in PCSC, we observed that inhibition of 5-Lox downregulates PKCε and phosphorylation of its substrates, but does not affect Akt or ERK in the same experimental conditions, suggesting that PKCε mediates the survival-regulating effects of 5-Lox in PCSCs (Figure 6A). We also observed that inhibition of 5-Lox decreases the protein-levels of survivin, cyclin D1, CDK4 and Bcl-xl, which are well-known regulators of cell survival and proliferation (Figure 6B).

### Inhibition of 5-Lox blocks *in vitro* invasion as well as soft-agar colony formation by prostate cancer stem cells

**Figure 7:**
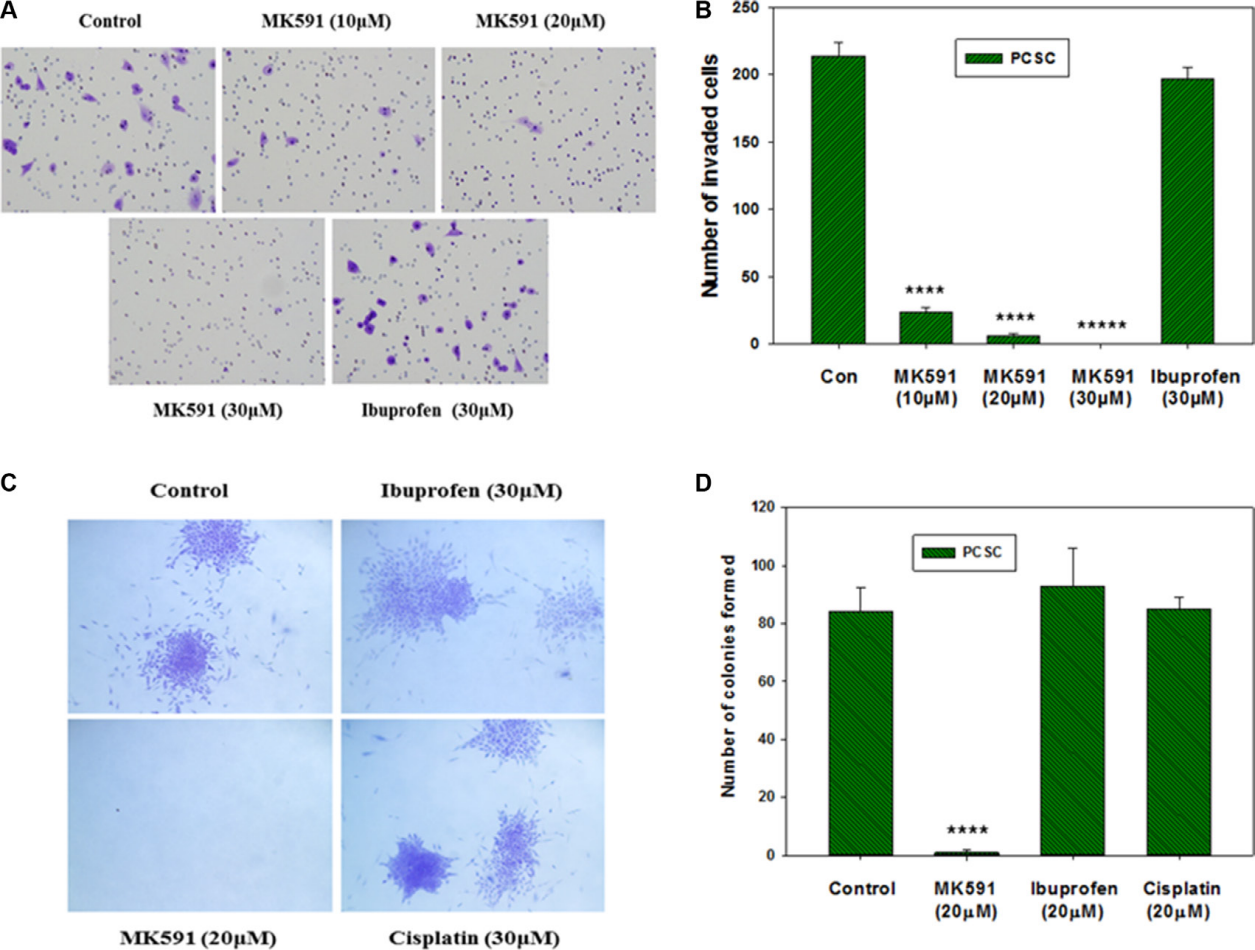
Effects of MK591 on *in vitro* invasion and soft-agar colony formation by PCSC In (**A**), invasive capabilities of PCS cells were assayed using matrigel-coated transwell chambers as described in the “Methods” section. After incubation, cells were fixed and stained with crystal violet. Pictures were taken with a Leica microscope at ×200. (**B**) Shows quantitative measurements of the number of invaded cells with or without drug treatment. Results represent mean values of individual data point ± standard deviation (*n* = 3). ^****^p = < 0.00005; ^*****^p = < 0.000005. In (**C**), effects of MK591 on soft-agar colony formation by PCSC are shown. Cells were plated on soft-agar in complete medium and treated with drugs as indicated. After incubation for three weeks, cells were stained with crystal-violet and growing colonies were counted under microscope at ×150. Note: Dramatic inhibition was observed with MK591 treatment whereas the effects of ibuprofen and cisplatin were not distinguishable. In (**D**), results are shown quantitatively as mean values of each data point ± standard deviation (*n* = 3). ^****^p = < 0.00005.

**Figure 8:**
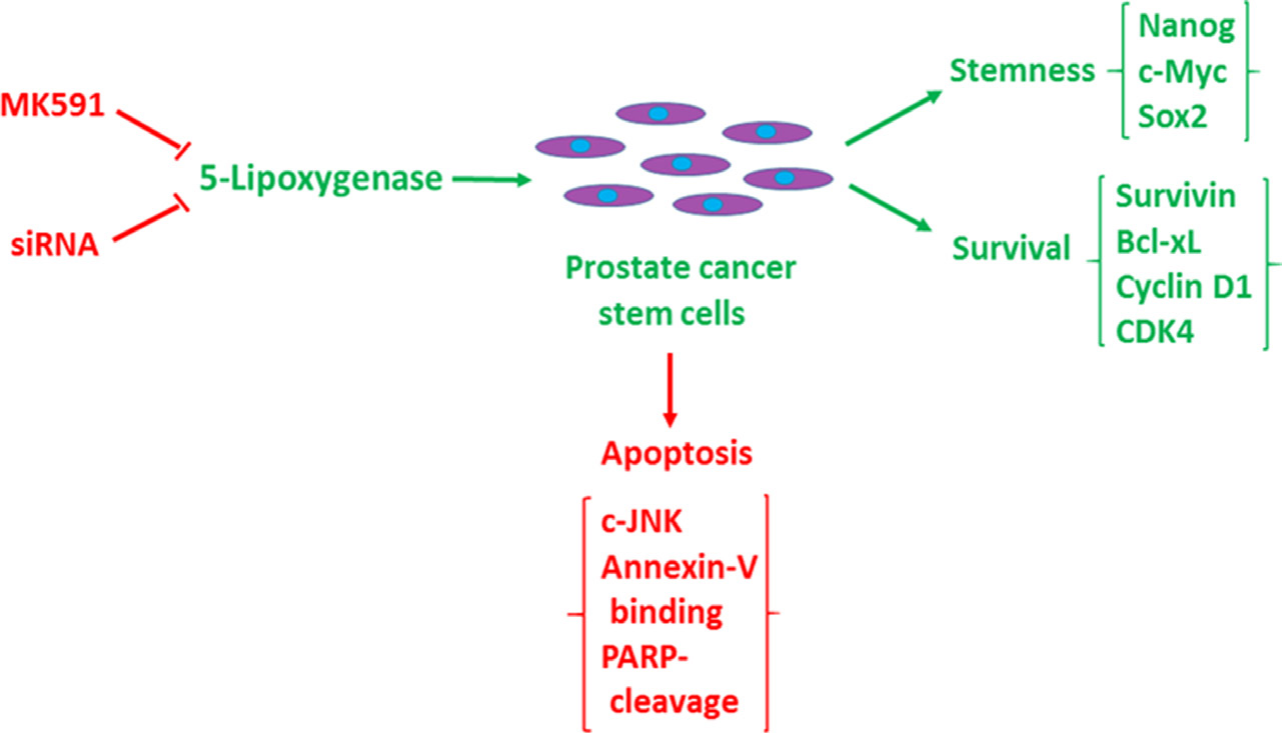
Diagrammatic representation of the role of 5-lipoxygenase in the maintenance of stemness and survival of prostate cancer stem cells Prostate cancer stem cells maintain stemness and tumorigenicity markers (Nanog, c-Myc, Sox2, CD44, CD133, ALDH1, ABCG2), and survival/proliferation markers (survivin, cyclin D1, CDK4, Bcl-xl) (Green), but undergoes c-JNK-mediated apoptosis when 5-lipoxygenase is inhibited (Red).

We observed that treatment with sub-lethal doses of MK591 (10–20 μM) dramatically decrease the *in vitro* invasion of prostate cancer stem cells through extracellular matrix (Figure 7A, 7B). Moreover, we found that MK591 dramatically affects the anchorage-independent colony-forming ability of prostate cancer stem cells on soft-agar (Figure 7C, 7D). Note: Ibuprofen and cisplatin were used in parallel experiments and found to be ineffective to block colony formation by prostate cancer stem cells in the same culture conditions, suggesting that the effect of 5-Lox inhibition in these processes is highly selective. These *in vitro* experiments indicate that the activity of 5-Lox is important for the maintenance of stemness and survival of PCSCs, and suggest that it is possible to inhibit the tumor-forming ability and therapeutic-resistance of PCSC by targeting 5-Lox with suitable agents (Figure 8).

## DISCUSSION

Our findings, for the first time, document that 5-Lox plays an essential role in the survival of prostate cancer stem cells, and that inhibition of 5-Lox kills these cells via induction of c-JNK-mediated apoptosis. Our observation of the massive induction of apoptosis in prostate cancer stem cells by specific inhibition of 5-Lox, opened up a unique possibility that both the progression and recurrence of prostate tumors can be vertically checked by eliminating these self-perpetuating and pluripotent cells by specific inhibitors of 5-Lox, such as MK591 (Figures 1, 2). We found that the prostate cancer stem cell subpopulation overexpress stem cells markers such as Nanog, c-Myc and Sox2 which play important roles in cancer stemness signaling. Interestingly, protein levels of these factors and sphere-forming abilities of PCSCs are severely down-regulated when the cells are treated with 5-Lox inhibitors (Figure 3), which suggest that the expression and function of the stemness factors in prostate cancer stem cells are dependent on 5-Lox activity. An important role of Myc has been characterized in cancer stem cells, and the formation of spheres in low-attachment plates is a confirmative test for cancer-stemness [41–44]. Moreover, loss of 5-Lox activity triggers mitochondrial permeability-transition and induces apoptosis in these cells (Figure 4). It has been characterized that CSCs are highly prolific and considerably more resistant to conventional chemotherapeutics (such as, cisplatin, paclitaxel, adriamycin, and methotrexate) and radiation, meaning while common therapeutic procedures eliminate majority of actively proliferating cancer cells and yield bulk tumor shrinkage, the population of CSC survive because of their relatively slow growth and aberrant activation of signaling pathways. However, we observed that inhibition of 5-Lox commits these cells to undergo self-killing via phosphatidylserine externalization, and cleavage of PARP protein. Moreover, we observed that 5-Lox inhibition-induced apoptosis in PCSC is mediated via activation of c-Jun N-terminal Kinase (Figure 5). Thus, we hope that agents such as MK591 may constitute valuable tools to exhaust the options of survival of these stubborn cells with virtually infinite self-renewing and incessant proliferating capabilities.

Though the 5-Lox activity appears to play an important role in the survival of PCSC, downstream mechanism through which 5-Lox delivers survival signaling is not clearly understood. We found that inhibition of 5-Lox triggers apoptosis in PCSC without inhibition of PI3K-Akt, or MEK-ERK, two well-characterized pro-survival mechanisms, but dramatically decreases the protein level of protein kinase C-epsilon (PKCε). These findings suggest that downstream mechanisms which mediate the survival-promoting effects of 5-Lox metabolites in PCSC involve the activity of PKCε, but not the more prevalent Akt or ERKs (Figure 6). Cancer stem cells possess the ability to disseminate and adhere to distant sites while retaining their stemness characteristics and thus regrow the tumor mass at new sites which eventually gives rise to metastatic lesions eventually causing demise of the affected patients. Though CSCs constitute only a minor population in a tumor, they are extremely potent to generate the heterogeneous lineages of cancer cells that make up bulk of the tumor, signifying the importance of identifying robust agents which can broadly affect and eliminate the metastatic ability of CSC. Interestingly, we found that MK591 strongly inhibits the *in vitro* invasion and completely blocks the anchorage-independent colony forming abilities of PCSCs on soft-agar at sub-lethal doses (Figure 7). Previously we have demonstrated that in standard culture condition human prostate cancer cells continuously generate metabolites of 5-Lox from arachidonic acid which play an essential role in their survival [23–26]. Blocking 5-Lox activity triggers rapid apoptosis in prostate cancer cells which can be effectively prevented by exogenous active metabolites of 5-Lox, 5(S)-HETE and its dehydrogenated derivative 5-oxoETE, documenting a pro-survival role of these metabolites in prostate cancer cells. The findings from our current *in vitro* experiments indicate that inhibition of 5-Lox triggers apoptosis also in prostate cancer stem cells, suggesting that 5-Lox activity greatly contributes to the survival and apoptosis-resistance of prostate cancer stem cells by metabolic conversion of arachidonic acid, and signaling via a mechanism which is similar to that what exists also in differentiated prostate cancer cells.

CSCs are a rare sub-population of cells present within the tumor, and are responsible for tumor-initiation, maintenance and tumor-recurrence. They possess unique biological properties, such as self-renewal and pluripotency. Moreover, they are thought to be more resistant to conventional chemotherapy and, as a result, are responsible for disease relapse. The core principle of cancer stem cells suggests that somewhere at the heart of a tumor there lies a handful of aberrant, genetically altered cells that are critical to maintain the malignant characteristics. This idea bears the potential to explain why tumors often regenerate long-time after even being destroyed by standard anticancer regimen. Moreover, it points to an alternative strategy for developing new generation of anticancer drugs, suggesting that these agents should be more selective towards the lethality of cancer stem cells and not to kill just any cells to shrink the tumor mass. Interestingly, cancer stem cells share many characteristics with normal stem cells, which include self-perpetuation and differentiation to other cells, and with growing evidence that cancer stem cells indeed exist in a wide variety of tumor types, it is changing the landscape of tumor biology research. It is also becoming increasingly relevant to understand the basic molecular mechanisms that regulate their self-renewal and differentiation because deregulation of genes involved in these pathways likely participate in basic characteristics of cancer *viz* tumor growth, recurrence and metastatic spread. The important question now is how to eliminate the cancer stem cells without killing normal stem cells because they are vital for maintaining tissue homeostasis? Since the inexorable growth of tumor is maintained by this rare group of stem/progenitor cells within it, we can expect that the cancer stem cells, because of their unique biological features, may be more dependent on certain cellular processes than normal cells and thus, will be more vulnerable to some agents that block those processes. However, sorting out unique pathways that are critical for cancer stem cells, but not for non-tumor cells, though of utmost importance, is incredibly complex.

The stem cell concept is enticing because of the potential it can provide to more durable and widespread cancer cures by identifying and attacking the tumor's most notorious cells. Evidence is slowly accumulating to document that prostate cancer stem cells/tumor-initiating cells are key drivers in initiation and progression of prostate tumor. Therefore, successful therapy must eliminate these cells, which is hampered by their high inherent resistance to common therapeutic modalities. The discovery of cancer stem cells has led to formulation of new strategies in future therapy of prostate cancer. The so called “cancer normal cells” can be eliminated by conventional therapies (surgery, radiation, chemotherapy) relatively easily, though these are rarely successful in attacking “cancer stem cells.” To be successful, the new approach should therefore be to develop therapeutic approaches to destroy these cancer stem cells. Thus far, only a handful of studies have addressed the cancer stem cell killing potential of standard apoptosis-inducing therapies and the underlying molecular mechanisms of apoptosis-resistance in these cells. Though our knowledge of PCSCs and their microenvironment remains a relatively new field, our current observations of PCSC's critical dependence upon 5-Lox activity for survival, as well as for the maintenance of self-renewal markers, make this regulatory mechanism ripe for developing experimental agents for effective management of prostate cancer. However, though it may be of high significance for development of durable prostate cancer therapy, whether 5-Lox activity plays any role in the survival of prostate cancer stem cells was never addressed before. This report documents that inhibition of 5-Lox, triggers rapid and wide-spread apoptosis in prostate cancer stem cells via activation of c-Jun N-terminal Kinase, and that this process is associated with down-regulation of PKCε, but not Akt or ERKs as we observed in regular prostate cancer cells [23–26, 37]. Moreover, inhibition of 5-Lox induces dramatic loss of stemness factors which are known to play important roles in self-regeneration and pluripotency of cancer stem cells. These findings indicate that 5-Lox activity may contribute to the survival and apoptosis-resistance of prostate cancer stem cells by metabolic conversion of arachidonic acid (an omega-6 polyunsaturated fatty acid, plentiful in our diets) and activation of PKCε, a transforming oncogene, and suggest that targeting 5-Lox with suitable agents may yield clues about effective, long-lasting therapy of prostate cancer and prevention of prostate cancer recurrence.

## MATERIALS AND METHODS

### Cell culture and reagents

Well-characterized human tumor-derived prostate cancer stem cells (CD133^+^, CD44^+^, ALDH1^+^, ABCG2^+^, Nanog^+^, c-Myc^+^) were purchased from Celprogen, Torrance, CA, USA [7] and were cultured in medium provided by the supplier. CD133^+^ human cord blood stem cells were isolated by Dr. N. Varma from fresh cord blood (provided by the Henry Ford Hospital) using antibody-coated magnetic beads (Miltenyi Biotech, San Diego, CA, USA). Human foreskin fibroblasts (HFF) and DU-145 prostate cancer cells were purchased from American Type Culture Collection (Manassas, VA, USA) and were grown in RPMI or DMEM medium 1640 (Invitrogen, Carlsbad, CA, USA). All the media were supplemented with 10% FBS and antibiotics. Antibodies against Nanog, c-Myc and survivin were purchased from R and D Systems (Minneapolis, MN, USA), and antibodies against, Sox2, CD44, CD133, ABCG-2, cyclin D1, CDK4, and Bcl-xl were from Santa Cruz Biotechnology (Santa Cruz, CA, USA). Polyclonal antibodies against Akt, phospho-Akt (Ser^473^), phospho-ERK1/2 (Thr^202/Y204^), phospho-JNK (Thr^183/Y185^), and phospho-Stat3 (Ser^727^), were purchased from Cell Signaling Technology (Danvers, MA, USA). Monoclonal anti-5-Lox antibody was purchased from BD Biosciences (Lexington, KY, USA). Anti-beta-actin antibody, ibuprofen and cisplatin were purchased from Sigma Chemical CO (St. Louis, MO, USA). MK591 was obtained as a generous gift from Dr. Robert N. Young (Merck-Frosst Centre for Therapeutic Research, Quebec, Canada).

### Cell viability assay

Cell viability was measured by MTS/PES One Solution Cell Titer Assay (Promega Corp, Madison, WI, USA) as described before [23–26].

### Microscopy

Cells (~3 × 10^5^) were plated in complete growth medium supplemented with 10% FBS overnight onto 60mm diameter tissue culture plates (Falcon) and allowed to grow for 48 hours. On the day of experiment, the spent culture medium was replaced with 2ml fresh RPMI medium and the cells were treated with inhibitors. Control cells were treated with solvent only (0.2% DMSO). Photographs were taken with a Nikon digital camera attached to a LEICA fluorescence microscope at ×400. Image acquisition and data processing were done with a Dell computer attached to the microscope using Q-Capture 7 software.

### Annexin-V binding

Cells (~3 × 10^5^) were plated in complete medium and allowed to grow for 48 hours. On the day of experiment, the spent culture medium was replaced with fresh 2ml medium and the cells were treated with MK591 or ibuprofen for 24 hours at 37°C. Then the cells were treated with FITC-labeled annexin-V and propidium-iodide for 15 minutes in the dark using an Annexin V-Binding Detection Kit following a protocol supplied by the manufacturer (BD Biosciences). After washing, cells were photographed using a Nikon digital camera attached to a LEICA fluorescence-microscope at 20×. Image acquisition and data processing were done with a Dell computer attached to the microscope using Q-Capture 7 software.

### Western blot

Cells (~3 × 10^5^) were plated in 60mm diameter tissue culture plates and allowed to grow for 48 hours. The old medium was then replaced with 2ml fresh medium and the cells were treated with inhibitors. After treatment, cells were harvested, washed, and lysed in lysis buffer (50 mM HEPES, pH 7.4, 150mM NaCl, 1mM EDTA, 1 mM orthovanadate, 10mM sodium pyrophosphate, 10 mM sodium fluoride, 1% NP-40, and a cocktail of protease inhibitors). Proteins were separated by 12% SDS–PAGE and transferred to nitrocellulose membranes. Membranes were blocked with 5% nonfat-milk solution and then blotted with appropriate primary antibodies followed by peroxidase-labeled secondary antibody. Bands were visualized by enhanced chemiluminescence (ECL) detection kit from Pierce Biotech (Rockford, IL, USA) and analyzed with a densitometer using Kodak imaging software. Unless otherwise mentioned, protein blots were analyzed in three independent experiments.

### Luciferase assay

PCS cells were transfected with lentiviral Nanog-luciferase constructs (> 90% cells transfected), expanded, and re-plated in 96 well culture plates in triplicates. Cells were then treated with MK591 or solvent only, and the luciferase activity was measured by a Luciferase Assay Kit from Promega Corporation (Madison, WI, USA) as we reported recently [25, 26]. Ibu (ibuprofen, a cyclooxygenase inhibitor) was used as negative control in parallel assays under the same culture conditions.

### Invasion assay

Invasion chambers (BD Biosciences) were soaked with 50 μl DMEM medium for 30 minutes at room temperature in cell culture hood. Then, a 50 μl single cell suspension of ~40,000 PCSCs in DMEM was gently placed on top of the matrigel with or without drugs. These chambers were then placed in twenty four well cell culture plates (one in each well) containing 500 μl of DMEM medium plus 2% serum as attractant. The chambers were incubated overnight in the incubator at 37°C. On the next day, matrigels inside the chambers were carefully scraped out and the membranes were fixed in methanol, stained with crystal violet, mounted on glass slides and counted under microscope.

### Soft-agar colony formation assay

Colony formation assays were performed in six well plates by placing ~5,000 Cells in 0.5 ml of 0.3% soft-agar on top of a 2 ml base layer of 0.6% agar. Plates were allowed to settle and then the wells were covered with 2 ml fresh medium containing 10% FBS with or without inhibitors. Plates were incubated at 37°C in the CO_2_ incubator for a maximum period of three weeks. Cell growth medium and inhibitors were exchanged every fourth day. At the end of incubation, cells were stained with 0.25% crystal violet and colonies were counted under a Leica microscope at ×150.

### Measurement of DNA degradation

Apoptosis was quantitatively measured by detecting degradation of nuclear DNA to nucleosomal fragments by sandwich-ELISA. Cells (~3 × 10^5^) were plated in 60 mm dishes and allowed to grow for 48 hours. Cells were then treated either with the experimental agents or the solvent vehicle for varying periods of time up to 24 hours. At the end of incubation periods, cells were lysed and the degradation of chromatin DNA to nucleosomal fragments was measured by Cell Death Detection ELISA^plus^ as described before [25, 26], following instructions supplied by the manufacturer (Roche, Indianapolis, IN, USA).

## CONCLUSIONS

Our findings revealed a new mechanism in the regulation of stemness and survival of prostate cancer stem cells, which suggests that by blocking 5-lipoxygenase activity with suitable agents we can effectively eliminate prostate cancer's most-resistant cells for development of a curative therapy of clinical prostate cancer and to prevent prostate cancer recurrence.

## Abbreviations

PCSC: prostate cancer stem cells
CBSC: cord blood stem cells
PKCε: protein kinase C-epsilon)
5-lox: 5-lipoxygenase
5-oxoETE: 5-oxoeicosatetraenoic acid
PARP: poly-ADP ribose polymerase
ELISA: enzyme-linked immunosorbent assay
FITC: fluorescein isothiocyanate
